# Depth Camera-Based 3D Hand Gesture Controls with Immersive Tactile Feedback for Natural Mid-Air Gesture Interactions

**DOI:** 10.3390/s150101022

**Published:** 2015-01-08

**Authors:** Kwangtaek Kim, Joongrock Kim, Jaesung Choi, Junghyun Kim, Sangyoun Lee

**Affiliations:** 1 Department of Electrical and Electronic Engineering, Institute of BioMed-IT, Energy-IT and Smart-IT Technology (Best), Yonsei University, 50 Yonsei-ro, Seodaemun-gu, Seoul 120-749, Korea; E-Mails: kwangtaekkim@yonsei.ac.kr (K.K.); ciyciyciy@yonsei.ac.kr (J.C.); jhkim_1012@yonsei.ac.kr (J.K.); 2 Future IT Convergence Lab, LGE Advanced Research Institute, 38 Baumoe-ro, Seocho-gu, Seoul 137-724, Korea; E-Mail: jurock.kim@lge.com

**Keywords:** 3D hand gesture tracking, 3D gesture control, tactile feedback, depth camera-based gestures, vision-based hand gesture interface, human computer interaction

## Abstract

Vision-based hand gesture interactions are natural and intuitive when interacting with computers, since we naturally exploit gestures to communicate with other people. However, it is agreed that users suffer from discomfort and fatigue when using gesture-controlled interfaces, due to the lack of physical feedback. To solve the problem, we propose a novel complete solution of a hand gesture control system employing immersive tactile feedback to the user's hand. For this goal, we first developed a fast and accurate hand-tracking algorithm with a Kinect sensor using the proposed MLBP (modified local binary pattern) that can efficiently analyze 3D shapes in depth images. The superiority of our tracking method was verified in terms of tracking accuracy and speed by comparing with existing methods, Natural Interaction Technology for End-user (NITE), 3D Hand Tracker and CamShift. As the second step, a new tactile feedback technology with a piezoelectric actuator has been developed and integrated into the developed hand tracking algorithm, including the DTW (dynamic time warping) gesture recognition algorithm for a complete solution of an immersive gesture control system. The quantitative and qualitative evaluations of the integrated system were conducted with human subjects, and the results demonstrate that our gesture control with tactile feedback is a promising technology compared to a vision-based gesture control system that has typically no feedback for the user's gesture inputs. Our study provides researchers and designers with informative guidelines to develop more natural gesture control systems or immersive user interfaces with haptic feedback.

## 1. Introduction

Over the past few years, the demand for hand interactive user scenarios has been greatly increasing in many applications such as mobile devices, smart TVs, games, virtual reality, medical device controls, the automobile industry and even in rehabilitation [1–8]. For instance, operating medical images with gestures in the operating room (OR) is very helpful to surgeons [9], and an in-car gestural interface minimizes the user's distraction while driving [10]. There is also strong evidence that human computer interface technologies are moving towards more natural, intuitive communication between people and computer devices [11]. Because of this reason, vision-based hand gesture controls have been widely studied and used for various applications in our daily life. However, vision-based gesture interactions are facing usability problems, discomfort and fatigue, which are primarily caused by no physical touch feedback while interacting with virtual objects or with computers with user-defined gestures [12]. Thus, co-locating touch feedback is imperative for an immersive gesture control that can provide users with more of a natural interface. From this aspect, developing an efficiently fast and accurate 3D hand tracking algorithm is extremely important, but challenging, to achieve real-time, mid-air touch feedback.

From a technical point of view, most of the vision-based hand tracking algorithms can largely be divided into two groups: model-based or appearance-based tracking. The model-based methods use a 3D hand model whose projection fits the obtained hand images to be tracked. In order to find the best fit alignment between the hand model and hand shapes in 2D images, optimization methods are generally used, which tends to be computationally expensive [13–20]. On the contrary, appearance-based methods make use of a set of image features that represent the hand or fingers without building a hand model [21–25]. Methods in this group are usually more computationally efficient than model-based methods, though this depends on how complex feature matching algorithms are used.

In regards to camera sensors used for tracking, there are also two groups: RGB or depth camera sensor-based methods. Until 2010, when the Kinect was first introduced, RGB camera-based methods were actively developed in the struggle with the illumination problem. Afterwards, depth sensors were widely used for hand tracking, due to their strength against illumination variation [26–28]. However, the previous systems with depth sensors are not sufficiently fast or accurate for the immersive gesture control that we are aiming to develop. Therefore, we developed a novel hand gesture tracking algorithm that is suitable to combine with tactile-feedback.

As mentioned earlier, adding haptic feedback to existing mid-air gestural interface technologies is a way of improving usability towards natural and intuitive interactions. In this regard, the first work that combined the Kinect-based hand tracking and haptic feedback was introduced a few years ago [29]. The developed system allows users to touch a virtual object displayed on a PC monitor within a limited workspace coupled with a pair of grounded haptic devices. Although it was not aimed at mid-air gestures with bare hands, it showed a feasible direction by showing an example using haptic feedback for hand tracking with a Kinect sensor. Our haptic feedback technology is in the same direction, but focuses on an add-in tactile feedback technology optimized for mid-air gesture interactions.

In this paper, our goal is to develop a novel gesture control system that provides users with a new experience of mid-air gesture interactions by combining vision-based tracking and wearable lightweight tactile feedback. To achieve the goal, four steps have been taken. First, we developed a new real-time hand gesture tracking algorithm with a Kinect sensor. The performance of the vision-based hand tracking system was measured in terms of accuracy and speed, which are the most important to consider in combination with tactile feedback. Second, a prototype of high definition (HD) tactile feedback was built with a piezoelectric actuator, so that any audio signals up to 6 KHz can be driven to display HD tactile feedback with ignorable delay. The prototype was mechanically tuned with a commercial driver circuit to provide strong tactile feedback to the user's hand. Third, a complete gesture control system was developed by integrating the tactile feedback technology into the hand tracking algorithm. Additionally, DTW (dynamic time warping) [30], the most well-known method in terms of speed and accuracy, was implemented and integrated for an immersive gesture control with tactile feedback, which is our goal. Last, the integrated system, the vision-based hand tracking combined with gesture recognition and tactile feedback, was systematically tested by conducting a user study with cross-modal conditions (haptic, visual, aural or no feedback condition) for four basic gestures. The evaluation results (accuracy, efficiency and usability) with the integrated system were analyzed by both quantitative and qualitative methods to examine the performance compared to the typical gesture interaction system, which is the case with no feedback.

The remainder of this paper is organized as follows. In Section 2, we describe how we developed a novel MLBP (modified local binary pattern)-based hand tracking algorithm with a Kinect sensor with the experimental results. Section 3 presents a new tactile feedback technology with a piezoelectric actuator that is not only simple to attach to the user's hand, but that is also integrable with any hand tacking system, followed by a proposal of a complete gesture control system with tactile feedback. The evaluation results achieved with the integrated system are reported in Section 4, and conclusions and future work are provided in Section 5.

## 2. MLBP-Based Hand Tracking Using a Depth Sensor

Real-time processing and precise hand tracking/recognition are essential for natural gesture controls. Our goal is therefore to develop a fast and accurate hand tracking algorithm. In this section, we propose a new hand tracking algorithm by employing MLBP, which is an extended idea from local binary pattern (LBP). In the following, the theory behind the MLBP method is presented followed by our proposed MLBP-based hand tracking algorithm with the evaluation results.

### 2.1. Modified Local Binary Pattern in Depth Images

**Figure 1.**
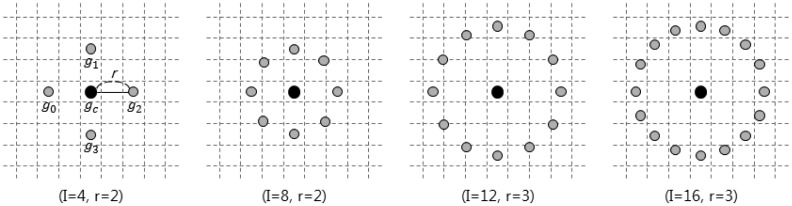
Modified local binary pattern with different *I* (the number of patterns) and *r* (the circle's radius) values.

LBP is the pattern of features, also called a texture descriptor, intensively used for classification with gray scale images. The MLBP that we propose is an effective approach to analyze shape information from depth images compared to the basic LBP methods [31,32]. Although the proposed MLBP is similar to LBP in that neighbor pixel values are thresholded by a center pixel value, it is specialized to accurately extract hand shape features from a sequence of depth images by adaptively estimating radius and threshold values depending on depth levels. MLBP consists of a number of points around a center pixel, and its radius is decided by the size of the target (hand) in depth images, as shown in Figure 1. On that account, MLBP can be mathematically represented as:

$$MLBP_{I,r}(x_{c},y_{c})=\sum_{t=0}^{I-1}s(g_{i}-g_{c})2^{i}$$

where (*x_c_*, *y_c_*) is the center position of a local window and *g_c_* and *g_i_*(*i* = 0, …, *I* − 1) denote the pixel values of the center point and the *i*-th neighbor point surrounding the center point, respectively. *r* is the radius of the circle, *I* is the number of patterns, and *s*(*z*) represents a thresholded value by $\{\begin{array}{l} 1,z≧threshold\\ 0,z<threshold \end{array}$ . Since the pixel values of a depth image represent real distances between objects and the sensor, different shape features can be extracted from depth images according to different thresholds. For example, when a distance threshold is 30 cm, all features at a depth of more than 30 cm from the sensor can be extracted by MLBP.

To achieve rotational invariance, each MLBP binary code must be transformed to a reference code that is generated as the minimum code value by the circularly bit shifting. The transformation can be written as:

$$MLBP_{I,r}=\text{min}\{ROR(MLBP_{I,r},k)|k=0.1\ldots .,I-1\}$$

where the function *ROR(x*, *i)* performs a circular bitwise right shift *i* times on the *I*-th binary number x. The *ROR(x*, *i)* operation is accordingly defined as follows:

$$ROR(MLBP_{I,r},K)=\sum_{i=k}^{I-1}s(g_{i}-g_{c})2^{i-k}+\sum_{i=0}^{k-1}s(g_{i}-g_{c})2^{I-k+i}$$

**Figure 2.**
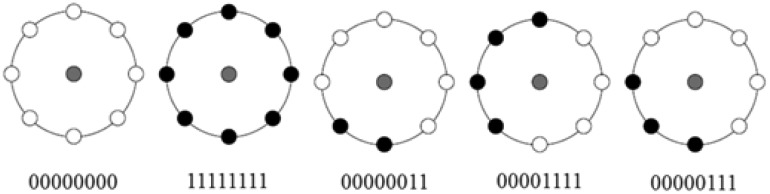
Some results of the modified local binary pattern (MLBP): White and black circles represent zero and one binary patterns, respectively

Figure 2 shows some results of MLBP as binary patterns.

### 2.2. A Proposed Hand Tracking Algorithm Using MLBP

Since a depth image does not contain texture and color information, it is difficult to detect and trace an object without such information. Using the proposed MLBP, we can precisely extract the shape of a target object in depth images in real-time. In this study, we apply the proposed MLBP to detect and track the position of hands in live depth images. Our proposed hand tracking system can be divided into two steps; hand detection and hand tracking. In the first step, the initial position of a hand to be tracked is detected. In the second step, robust hand tracking is performed with the detected hand's position. From a technical point of view, the details of the algorithms are provided in the following.

#### 2.2.1. MLBP-Based Hand Detection in Depth Images

**Figure 3.**
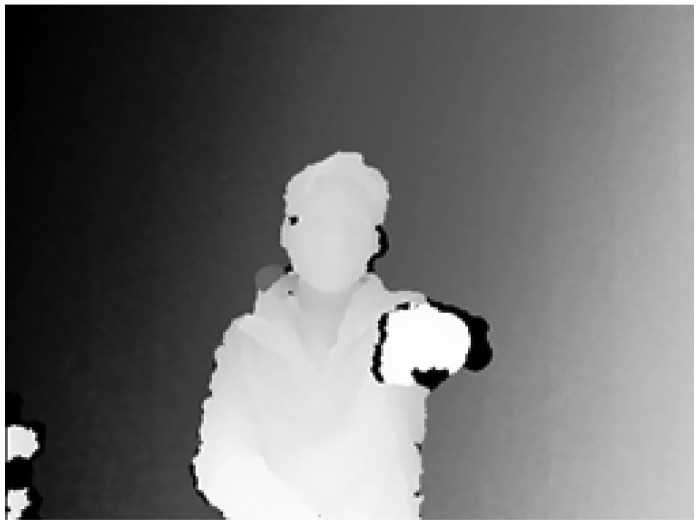
Arm extension motion to initialize the hand detection process.

**Figure 4.**
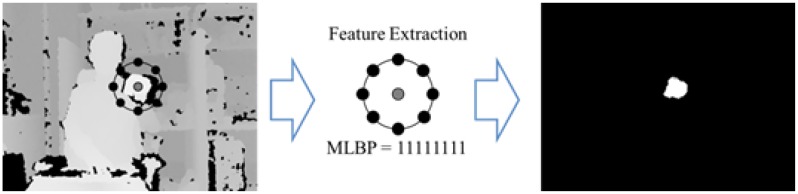
The resulting image of the MLBP with a threshold of 30 cm.

To detect the initial position of a hand, we use the arm extension motion with a fist towards the sensor as an initializing action. For that reason, we need to extract the fist shape in the depth images using the proposed MLBP, as shown in Figure 3. To extract fist shape features in depth images, we assume that there is no object detected near the hand within 30 cm when a user stretches forward with his/her hand in front of the sensor. Therefore, all of the binary values of MLBP with a threshold of 30 cm should be “1's which form hands” candidates, as shown in Figure 4. Finally, we search all position of hand candidates in the depth images and decide the initial position of a hand that is detected continuously at the same location with the previous five frames.

#### 2.2.2. MLBP-Based Hand Tracking in Depth Images

**Figure 5.**
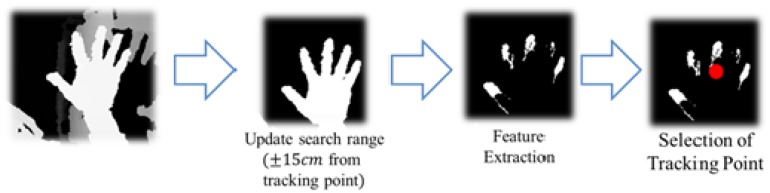
Overview of the proposed hand tracking algorithm.

**Figure 6.**
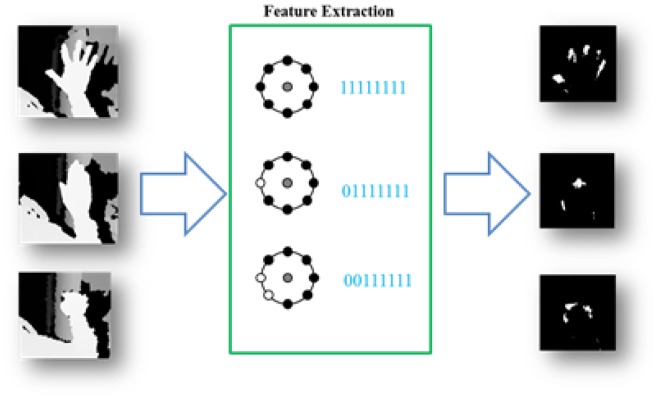
Example results of hand feature extraction using MLBP with a threshold of 10 cm.

With the initially-detected hand position, hand tracking is performed to estimate and track the hand's location rapidly and precisely. The hand tracking can be divided into three steps, as shown in Figure 5: (1) updating a search range; (2) extracting hand features; and (3) selecting a tracking point. As the first step, we need to define a decent search range for a fast estimation of hand locations. The search ranges in x- and y-coordinates are set to six-times bigger than the hand size in depth images based on a pilot experiment, and an acceptable distance range for the z-coordinate is set to ±15 cm. In the feature extraction step, hand-feature points are extracted by MLBP within the search range. When the threshold of MLBP is set to 10 cm, the number of the “0” values in MLBP becomes less than or equal to I/4, where *I* is the number of patterns, as shown in Figure 6. The last step is a process to determine a point to be continuously tracked from the extracted feature points. For this step, the center location of the extracted points is computed first, and then, the nearest feature point from the center is chosen as the tracking point. This way, we can avoid the risk of tracking outside the hand region. As long as the hand tracking is not terminated, Steps 1 through 3 are continuously repeated.

### 2.3. Experimental Results

**Figure 7.**
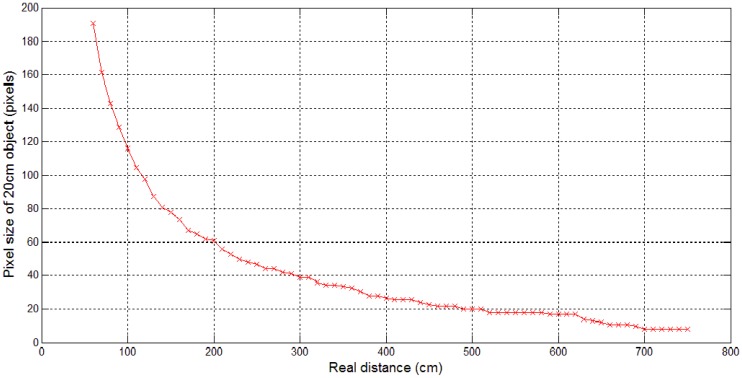
Object size variations measured in a pixel with a rectangular object (20 cm wide) in depth images at different distances from 60 cm to 750 cm.

Our proposed MLBP hand tracking offers real-time and accurate hand tracking, which is suitable for a real-time gesture control system with tactile feedback. In order to verify the hand tracking system, several experiments have been conducted to measure the performance in terms of computational time and accuracy We used a Kinect depth sensor capturing VGA (640 × 480), RGB and depth images at 30 fps. The data acquisition was implemented in the Open Natural Interaction (OpenNI) platform, while other modules were implemented using C on a Windows machine with a 3.93-GHz Intel Core i7 870 and 8 GB RAM. The number of MLBP patterns has been set to 16, since this showed the best performance in terms of tracking accuracy and processing time by a pilot experiment. It is suggested that the radius of MLBP be adaptively chosen, because the object size in a depth image varies from distance to distance, as shown in Figure 7. Based on the measured data, we were able to adaptively choose radius values according to the distance (see Table 1). Those radius values were used for the following evaluation experiments.

**Figure 8.**
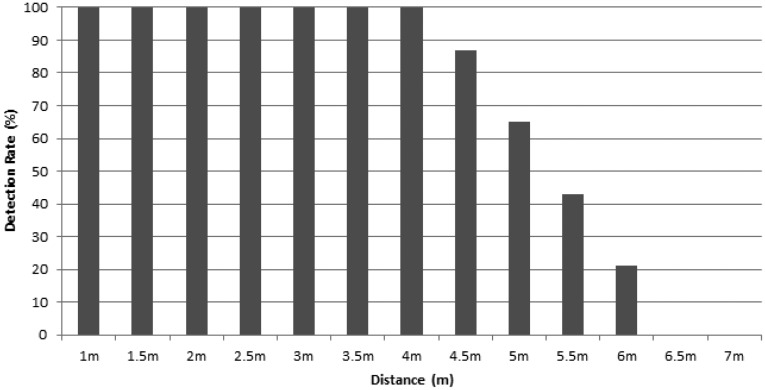
Detection rate according to a distance from 1 to 7 m.

As the first evaluation experiment, the accuracy of hand detection was tested from 1 m to 7 m at 50-cm intervals. Detection rates were computed by taking the average of 2000 attempts from 100 people. Figure 8 shows the detection rates over several distances. As clearly observed on the plot, the detection rate is kept perfect until reaching 4 m and, thereafter, rapidly drops to 6 m, mainly due to deteriorated depth images. It was also learned that the hand size becomes too small to be recognized when the distance exceeds 4.5 m. Therefore, we preferably chose a workspace from 1 m to 4 m for our work (detection and tracking), since this provides most reliable depth images.

In the second experiment, we focused on verifying our hand tracking algorithm by comparing other state-of-the-art hand tracking methods listed below:

- PrimeSense's Natural Interaction Technology for End-user (NITE)
- A color information-based object tracking technique (CamShift) [33]
- 3D hand tracking using the Kalman filter (3D Tracker) [34]

**Figure 9.**
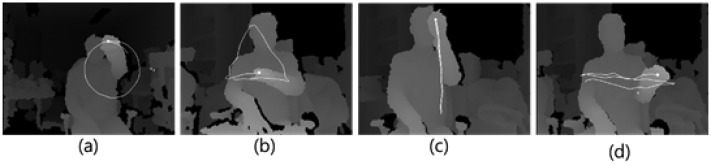
Four gestures used for the hand tracking evaluation: (**a**) circle; (**b**) triangle; (**c**) up to down and the reverse; (**d**) left to right and the reverse.

We chose the three methods for the evaluation because: (1) the CamShift algorithm is a well-known tracking method for color images; and (2) NITE and 3D Tracker are considered the most advanced tracking technologies for depth images. To verify the robustness of our proposed hand tracking under different hand movements, we made a dataset based on 100 identities each with four gestures at different standing distances (1 m, 2 m and 3 m), as shown in Figure 9. For this experiment, the radius values of the MLBP used in the hand tracking algorithm for evaluation are listed in Table 2.

**Figure 10.**
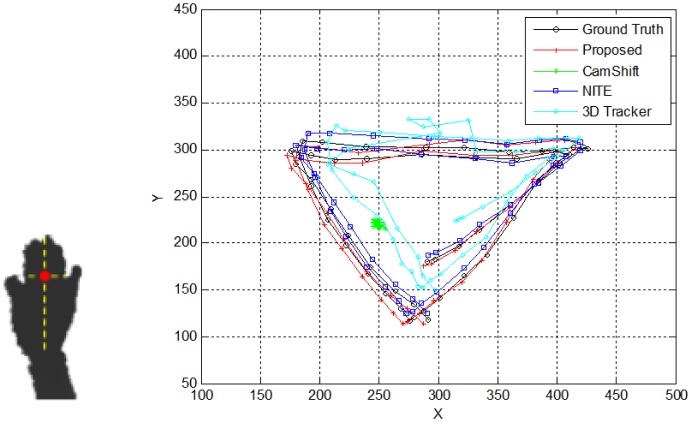
Ground truth (red dot) manually selected as one-third of the hand from the top **(Left)** and the measured trajectories by four methods for a triangle gesture **(Right).**

**Figure 11.**
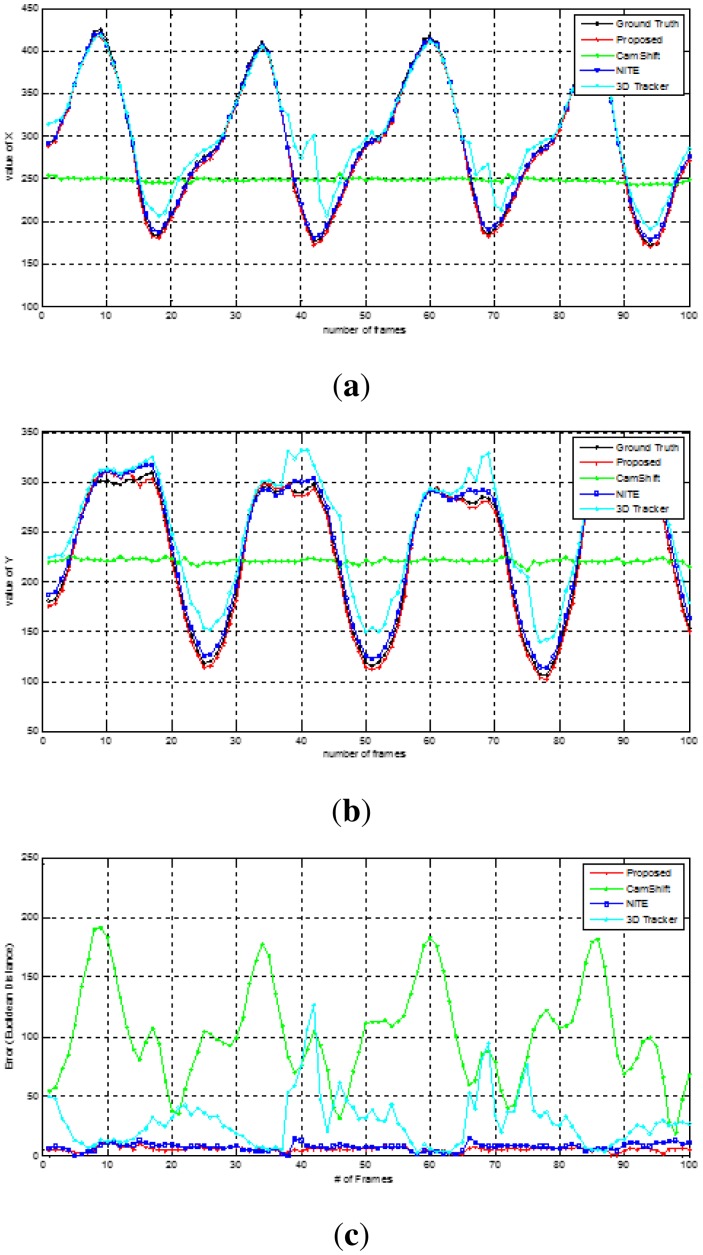
Comparisons of the tracking accuracy: (**a**) x-axis; (**b**) y-axis; and (**c**) RMS errors between the ground truth and the tracking position.

The ground truth for the evaluation was manually selected and marked by red, as shown in Figure 10. For the quantitative analysis, the geometric errors between the ground truth and the tracking position were measured at different distances (1 m, 2 m and 3 m) five times for each predefined hand movement with 100 people who voluntarily participated. The right image of Figure 10 shows tracking trajectories recorded in x,y-coordinates by the four tracking methods regarding a triangle gesture. Three methods, including our method, but 3D Hand Tracker, draw a clear and stable triangle shape close to the ground truth. A systematic analysis in terms of accuracy can be done by looking at the data in Figure 11. It is evident that the tracking trajectory only by our method accurately follows the ground truth on both the x- and y-axes, though NITE shows good performance, but not as precise as our method (see the RMS errors). The fact becomes more obvious when analyzing the numerical error data summarized in Table 3. Our proposed method outperforms the other three methods over all distances. Note that the averaged errors decrease as the distance becomes larger, because the variations of the hand's position in 2D images are reduced as the distance increases.

We conducted a further experiment with the predefined four gestures of Figure 9 to investigate the accuracy on real gestures, since our goal is to integrate our tracking method into a gesture control system. The numerical results of averaged errors are summarized with the standard deviation in Table 4 and confirm that our method still provides the best accuracy at tracking the four gestures in real time. Overall, the CamShift algorithm shows the worst tracking performance, since it relies heavily on color information, and tracking often fails when the user's hand moves close to the face, the other hand or skin-color-like objects. In addition, with the 3D Hand Tracker using the Kalman filter in depth images, the tracking is not as accurate as our method, because the tracking point is obtained based on the central point of an ellipse that encloses the hand detected by the initializing process. Our hand tracking algorithm runs at 28 ms (35 fps), 15 ms (66 fps) and 12 ms (83 fps) at 1 m, 2 m and 3 m, respectively, with a sequence of VGA input images. These results demonstrate that our proposed tracking method is the most accurate and sufficiently fast for a real-time haptic-assisted gesture control system, which is our next step in this study.

We summarize the results in Tables 1 and 2, which show the average RMS error and the standard deviations. Table 3 shows the results with respect to different distances (1 m, 2 m and 3 m), and Table 4 shows the results with respect to different hand gestures.

## 3. Development of Hand Gesture Control with Tactile Feedback

In this section, we present a new gesture control system incorporated into tactile feedback towards a real-time immersive gesture control system.

### 3.1. Prototyping a Wearable Tactile Feedback Device Using Piezoelectric Actuators

**Figure 12.**
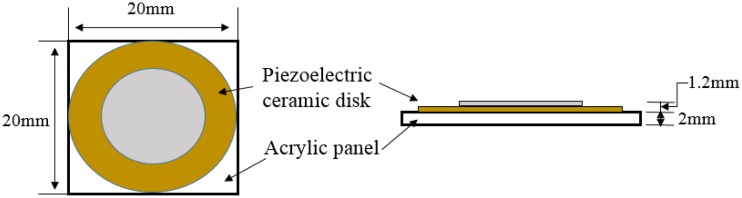
Haptic actuator designed for tactile feedback.

**Figure 13.**
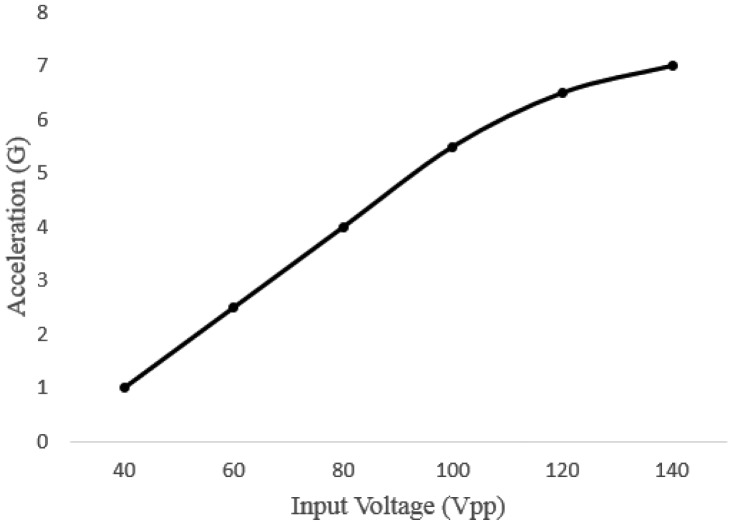
Performance (acceleration) measured with the designed haptic actuator *vs.* the input voltage.

A tactile feedback device was designed with a piezoelectric actuator, which precisely bends when a differential voltage (e.g., 10 to 200 Vpp, Voltage Peak-Peak, measured from the top to the bottom of the waveform) is applied across both ends, to provide haptic feedback for gesture control-based interactions. To develop a high definition (HD) tactile feedback device, a commercial piezoelectric actuator (Murata Manufacturing Co., Ltd. 25 mm diameter; see Figure 12) that converts an electric signal into a precise physical displacement was utilized. For our design, the piezoelectric actuator was affixed to a transparent acrylic square (20 mm long and 2 mm thick), since it plays the roles of an electrical insulator and a booster, enhancing vibrations on the surface. The thickness of acrylic panel was determined as 2 mm after a pilot experiment measuring the strength of the tactile feedback versus the usability of the user's hand. Our goal was to minimize the thickness, but to maximize the strength of the haptic feedback, since it was learned that the thickness of the acrylic panel is proportional to the strength of the vibration. The final design of the haptic feedback actuator (weight, 3.7 g) is shown in Figure 12. An audio signal amplifier circuit (DRV 8662 EVM, Texas Instrument Inc.) was used for amplifying tactile signals and driving the designed haptic actuator. In this design, any tactile signal can be alternatively used for operating the haptic feedback device, as long as its frequency is lower than 6 KHz. To measure the performance of tactile feedback on the haptic actuator, acceleration was measured by an accelerometer (KISTLER 8688A50) with input voltage (one cycle of a saw tooth at 500 Hz) varying from 40 to 140 Vpp. As seen in Figure 13, tactile feedback strength linearly increases as the input voltage gets bigger. In order to decide the optimal strength of tactile feedback, we conducted a pilot study with a simple psychophysical method, the method of limit, to find a perceptual threshold at which a tactile stimulus can be detected 100% of the time by all participants. The found stimulus intensity on the palm was 3G (gravitational acceleration).

### 3.2. Development of a Mid-Air Gesture Control System with Tactile Feedback

**Figure 14.**
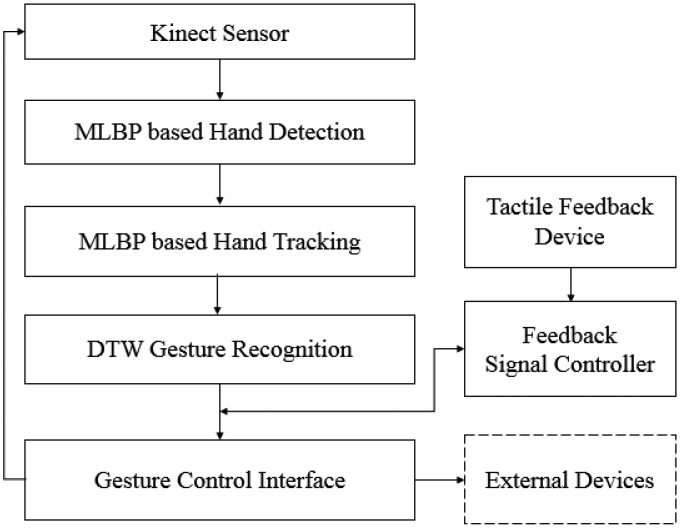
Block diagrams of the proposed mid-air gesture control system with tactile feedback.

As mentioned before, mid-air gestures suffer from more fatigue and are more error prone than traditional interfaces (e.g., the remote control and the mouse), due to the lack of physical feedback. Our goal is therefore to add tactile feedback to a real-time hand gesture tracking and recognition system. To achieve this, we integrated the developed real-time MLBP-based hand tracking system with a prototype of the hand-mountable tactile feedback. For gesture recognition, we exploited an existing algorithm, multidimensional dynamic time warping-based gesture recognition [30], which is well known as the best in terms of accuracy and speed, since in our application, real-time processing is crucial to provide simultaneous tactile feedback. The implemented gesture recognition algorithm was even further customized, so that the speed becomes the max, though results in a tolerable loss of accuracy (e.g., average 80%–85% for predefined gestures 18). Block diagrams of our developed system, including the in-out flow, are drawn in Figure 14. In the block diagrams, the method of incorporating the haptic feedback can be flexible with the user scenarios, though we focus on the feedback for gesture recognition. For instance, tactile feedback in our developed system is also synchronizable to hand detection, tracking and even usage warning by simple modifications with software programming.

Our developed mid-air gesture control system is efficiently fast (average 35 fps on a PC with a 3.4-GHz Intel Core i7-3770 CPU, RAM 16 GB), including detection, tracking, recognition and tactile feedback with an RGBD input image (320 × 240 pixels) from a Kinect sensor and provides accurate gesture recognition, although it varies from gesture to gesture. In regards to tactile feedback, predesigned tactile signals lower than 6 KHz are stored in local data storage and automatically sent to the feedback signal controller to drive the haptic actuator in response to a trigger signal controlled by the block of the gesture control interface. With our gesture control system, any external devices can be operated more accurately in real time, since it provides in-air touch feedback that will significantly improve usability in air gesture interactions. The evaluation results with our developed system are presented in the next section.

## 4. Evaluation of Hand Gesture Control with Tactile Feedback

A user study has been conducted to evaluate our haptics-assisted hand gesture control system in comparison with no feedback and the other two modalities (visual and aural feedback). The testing results were then analyzed by both quantitative and qualitative methods to verify the performance (accuracy, trajectory and speed), including usability. The testing results were quantitatively analyzed by ANOVA (analysis of variance). An in-depth qualitative analysis was also processed to inspect any improvement in the usability. In the following, the method of the user study and the experimental results are presented.

### 4.1. User Study for Evaluation

#### 4.1.1. Participants and Apparatus

**Figure 15.**
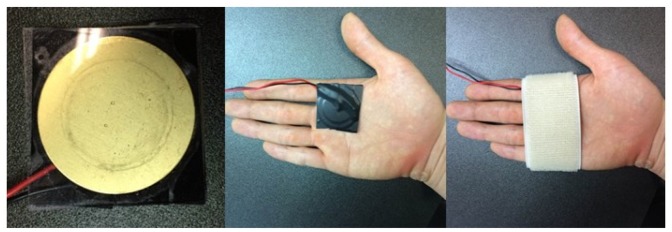
Haptic feedback device setup: a piezoelectric actuator glued on a transparent plastic panel **(Left)** and its attachment to the user's hand with an elastic bandage.

Six participants (five males and one female, aged from 26 to 31 years old) took part voluntarily in the experiment. All participants were right-handed and self-reported no visual nor haptic impairment. Three of the participants had previous experience with gesture-controlled systems. All but one participant had no experience with haptic interfaces. In the experiment, a standard PC monitor (“27” LED) and an earphone were used for visual and aural feedback, respectively. For haptic feedback, a tactile feedback prototype developed in the section above was used for mid-air gesture interactions. The haptic actuator was attached to the user's hand, as seen in Figure 15. The driver of the haptic actuator was connected to the main PC that runs the developed real-time hand tracking and recognition algorithms. Automatic triggering for feedback signals was encoded by software programming.

#### 4.1.2. Feedback Stimuli

**Figure 16.**
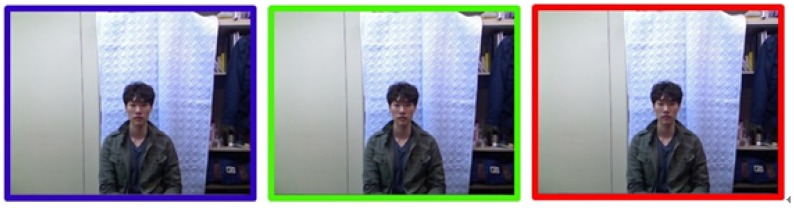
Three signals for the visual feedback: blue, gesture begin/end; green, success of recognizing a gesture; red, fail to recognize a gesture.

**Figure 17.**
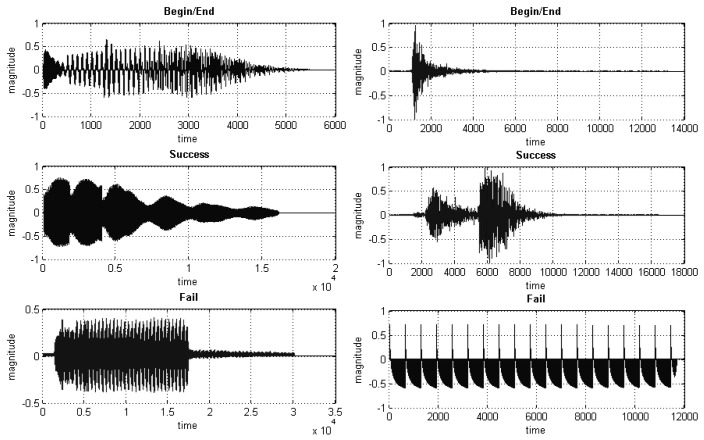
Three signals for the aural feedback **(Left)** and haptic feedback **(Right).** The unit of magnitude is dB.

After a series of pilot experiments in learning feedback locations and perceptual levels of feedback signals on the sensory modalities, three identifiable feedback signals were chosen for gestures' beginning/ending, gesture success and gesture failure in recognition. The designed feedback signals are shown in Figures 16 and 17 for visual, aural and haptic feedback, respectively. Those signals were pre-stored in the PC and triggered by the developed gesture interface control system.

#### 4.1.3. Conditions

**Figure 18.**
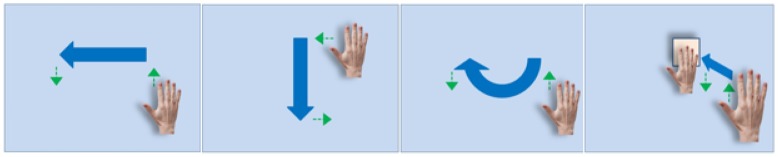
Four basic gestures designed for our study: The green arrows present the instructed motions to begin and end each gesture.

There were four experimental conditions: no feedback (NF), visual feedback (VF), haptic feedback (HF) and aural feedback (AF). For each condition, four hand gestures (right to left, up to down, half circle, push; see Figure 18) were tested to investigate the effect of feedback for mid-air hand gestures. We chose the four gestures, since those are commonly used for operating smart devices and are a basic set that can form more complex gestures. Each gesture per condition was repeated fifty times. The order of conditions with gesture types was randomized to avoid the learning effect.

#### 4.1.4. Procedure

**Figure 19.**
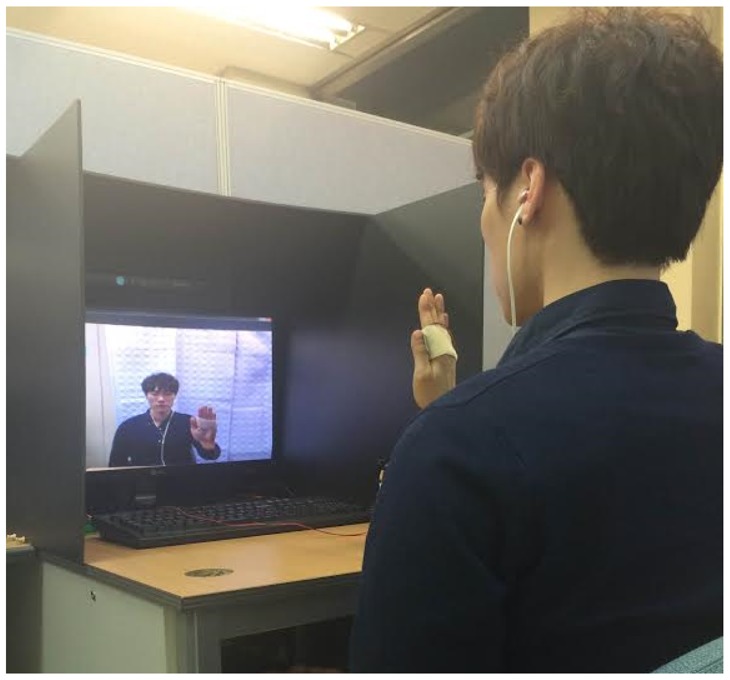
The experimental setup.

Prior to beginning the experiment, all participants took a training session until becoming familiar with the experimental procedure, which took about an average of 30 min, varying from person to person. In the main experiment, the participants were comfortably seated in front of a computer monitor, as shown in Figure 19. They wore ear phones to block noises for the visual and haptic feedback conditions or to hear audio sound for the aural feedback condition. Noise blocking was achieved by playing a white noise sound signal for the visual and haptic conditions. For the haptic condition, the developed haptic actuator was attached to the participant's hand by an elastic bandage, as shown in Figure 15. Each subject followed a randomized sequence of the four gestures per condition. For each condition, 50 trials (repetitions) for a gesture, split into five blocks, were collected for the quantitative data analysis. This resulted in 800 trials in total for each subject. To reduce learning effects, the order in which the runs are presented was also randomized and unknown to the participant. On each trial, one of the four gesture types (see Figure 18) was graphically displayed on top of the screen for the participant to easily follow a given gesture task in his/her most natural way. During the experiment, recognition rates, gesture trajectories and the elapsed times were recorded to measure the control system's performance and usability. After finishing the experiment, all participants were asked to fill out a standard NASA Task Load Index (TLX) questionnaire and a preference interview form for the qualitative data analysis. The participants were required to visit twice to complete all of the trials. It took an average of two and half hours for each participant to complete the whole experiment.

#### 4.1.5. Data Analysis

**Figure 20.**
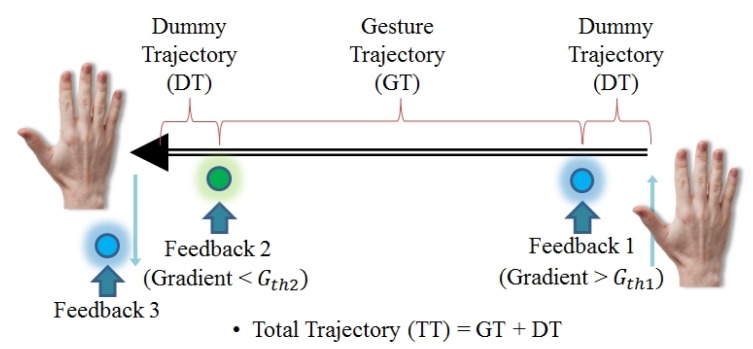
An example of gesture trajectories with the feedback locations in the right to left gesture.

For the quantitative evaluation with/without feedback, we developed three indexes: recognition rates, trajectories and the speed that can represent performance. Accuracy is measured by computing gesture recognition rates as the equation below for each gesture per condition. For example, in the experiment, recognition rates were computed every 10 trials and repeated five times for the statistical analysis. This metric involves investigating the effect of the provided feedback on the gesture recognition system. The other two indexes defined for our study are trajectories and speed, which may be correlated to usability. As illustrated in Figure 20, total trajectory (TT), gesture trajectory (GT) and dummy trajectory (DT, pre-gesture trajectory) are defined and used for inspecting how efficient hand gesture movements are with/without feedback. Note that users tend to move their hands more with the no feedback condition due to the unknown and invisible gesture tracks, which is a cause of fatigue. The longer trajectory is regarded as having lower efficiency in our evaluation. Gesture speed is also measured and was statistically analyzed to see the correlation with feedback. All metrics are defined by the equations below, and the results were statistically analyzed by ANOVA. Additionally, qualitative data from both a standard NASA TLX questionnaire and a preference rating form were also analyzed in comparison with the quantitative data.

$$\begin{array}{c} \text{Recognitionrate}\;\text{rate}=\frac{\#of\;\text{successful}\;\text{recognition}}{\#of\;\text{trials}}\\ TT=\overset{k}{\underset{i=1}{\text{∑}}}\left\{P_{i}(x,y,z)-P_{i-1}(x,y,z)\right\}\\ DT=\overset{l}{\underset{i=1}{\text{∑}}}\left\{P_{t}(x,y,z)-P_{t-1}(x,y,z)\right\} \end{array}$$

where *P_i_* is a point at the *t* image frame and *k* means the total number of frames for gesture recognition. *l* is the number of frames, which is determined by predefined gradients values.

$$\text{Speed}=\frac{TT}{\text{Time}\;\text{for}\;\text{each}\;\text{gesture}}$$

### 4.2. Experimental Results

In this section, the experimental results of the evaluation with our gesture control system are reported. The three indexes (recognition rate, trajectories, speed) measured through the experiment are shown as quantitative results. Participants' responses to the NASA TLX questionnaire and the preference rating form are summarized as qualitative results.

#### 4.2.1. Quantitative Evaluation

**Figure 21.**
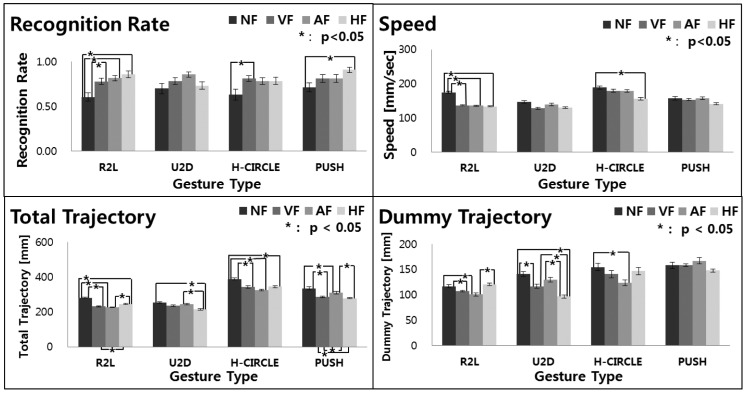
Quantitative evaluation results of the four hand gestures across four feedback conditions. The bar represents the average values of each metric obtained from the experiment, and the error bar shows the standard error. In all but the recognition rates, lower values are better. In the plots, NF, VF, AF and HF stand for no feedback, visual feedback, aural feedback and haptic feedback, respectively, and R2L, U2D, H-circle, and push represent right to left, up to down, half circle and push gestures, respectively.

We ran a one-way ANOVA to analyze the three indexes, recognition rate, trajectory and speed, with three feedback conditions, and the results are shown in Figure 21. In regards to recognition rate (accuracy), all gesture types, but the up to down gesture (*F*_3,1192_ = 2.53, *p* < 0.0604), showed significant differences with all feedback in right to left (*F*_3,1192_ = 9.07, *p* < 0.0001), with visual feedback in half circle (H-circle) (*F*_3,1192_ = 3.31, *p* = 0.0227) and with haptic feedback in push (*F*_3,1192_ = 3.92, *p* = 0.0104). The results clearly show that recognition rates with the no feedback condition were all lower than those with the other feedback conditions, and haptic feedback positively influenced the accuracy. A *post hoc* Tukey test revealed that in the right to left gesture, the recognition rates of all feedback conditions were significantly larger than those with the no feedback condition (NF *vs.* VF, *p* = 0.0064; NF *vs.* AF, *p* = 0.0006; NF *vs.* HF, *p* < 0.0001), while no difference was found among the feedback conditions with the up to down gesture (NF *vs.* VF, *p* = 0.5165; NF *vs.* AF, *p* = 0.0524; NF *vs.* HF, *p* = 0.9462).

Similarly, an ANOVA on the feedback conditions did show significant results on speed (right to left, *F*_3,11926_ = 120.66, *p* < 0.0001; up to down, *F*_3,1192_ = 2.53, *p* = 0.0604; half circle, *F*_3,1192_ = 3.31, *p* = 0.0227; push, *F*_3,1192_ = 3.92, *p* = 0.0104). Interestingly, the highest speed was achieved with the no feedback condition over all gesture types. A pairwise Tukey test did show significant differences in the right to left gesture for all feedback conditions (NF *vs.* VF, *p* < 0.0001; NF *vs.* AF, *p* < 0.0001; NF *vs.* HF, *p* < 0.0001) and in the half circle gesture for the no feedback and the haptic feedback conditions (*p* = 0.0023).

Regarding total trajectory (TT), the ANOVA test did show significant results across all gestures (right to left, *F*_3,1192_ = 120.66, *p* < 0.0001; up to down, *F*_3,1192_ = 34.76, *p* < 0.0001; half circle, *F*_3,1192_ = 21.50, *p* < 0.0001; push, *F*_3,1192_ = 18.74, *p* < 0.0001). Average values with the no feedback condition were all higher than those with the other conditions for all gestures. A Tukey test confirmed that all feedback conditions are significantly different, except the up to down gesture. We also observed significant results on the dummy trajectory (DT) on all feedback conditions (right to left, *F*_3,1192_ = 13.85, *p* < 0.0001; up to down, *F*_3,1192_ = 21.86, *p* < 0.0001; half circle, *F*_3,1192_ = 3.86, *p* < 0.0001; push, *F*_3,1192_ = 18.49, *p* < 0.0001). A Tukey test confirmed that the no feedback condition was significantly different from the other feedback conditions for the right to left gesture (NF *vs.* VF, *p* = 0.0093; NF *vs.* AF, *p* = 0.0005; NF *vs.* HF, *p* < 0.0001), the Up to Down (NF *vs.* VF, *p* = 0.0025; NF *vs.* HF, *p* < 0.0001; VF *vs.* HF, *p* = 0.0284; AF *vs.* HF, *p* < 0.0001) and the half circle (NF *vs.* AF, *p* = 0.0062). One clear pattern is that the longest trajectory is formed with the no feedback condition.

These results, the higher speeds and the longer trajectories with the no feedback condition indicate that users had to move their hands faster and longer than the other feedback conditions. Those behaviors are caused by no spacial cue for the gesture recognition, in comparison with the other feedback conditions, which actually help users virtually draw and memorize the spacial trajectories of gestures. In addition, the more accurate recognition rates were also achieved with the feedback conditions, because trajectory guidance feedback can provide users with a learning effect on the better gesture recognition.

#### 4.2.2. Qualitative Evaluation

**Figure 22.**
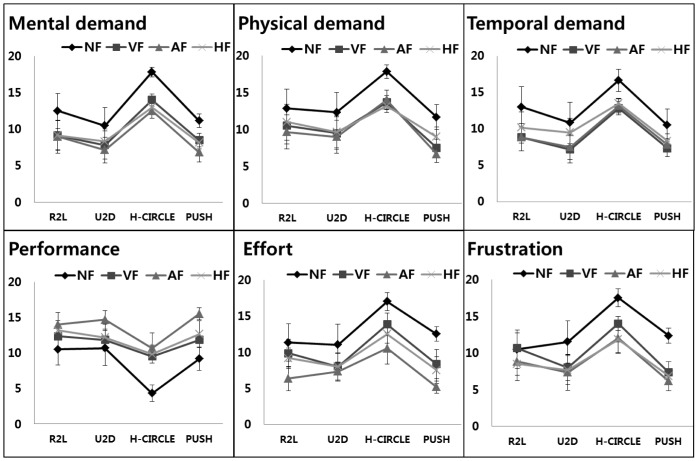
The participants' responses to the NASA-Task Load Index (TLX) questionnaire. In all but performance, a lower value is better.

After finishing the experiment, participants filled in the NASA Task Load Index (NASA-TLX) questionnaire in regards to their feelings about the experiment. The NASA-TLX questionnaire has six rating categories about feelings (mental demand, physical demand, temporal demand, performance, effort and frustration). Each scale of the TLX is divided into 20 equal intervals. We conducted this evaluation, because the quantitative results can be well interpreted as a user experience perspective, which will eventually show a correlation between feedback and fatigue. The results are shown in Figure 22. As expected, there are apparent differences between two groups (no feedback *vs.* feedback). The gap between the two groups is consistent over gesture types and workload categories. It corroborates that: (1) haptic is an effective way to reduce workload and to improve gesture performance; and (2) the no feedback condition causes relatively more fatigue no matter what the type of gestures for the mid-air interactions. The evidence is still valid even with more complicated gestures, like half circle, whose rates were the highest in the mental, temporal and physical demand categories. Based on this finding with the quantitative results, it is shown that the no feedback condition, resulting in the lower recognition rate, the faster speed and the longer trajectory, increases fatigue for mid-air gesture interactions, though we do not prove it by taking a physiological and biomechanical view, which will be conducted in the near future.

We additionally collected user's preference data on the feedback conditions. All participants answered the following questions: (1) In which feedback condition did you feel most pleasant? (2) In which feedback condition did you feel most comfortable? (3) Which feedback condition did you feel was most physically demanding? (4) In which feedback condition did you feel most frustrated?

**Figure 23.**
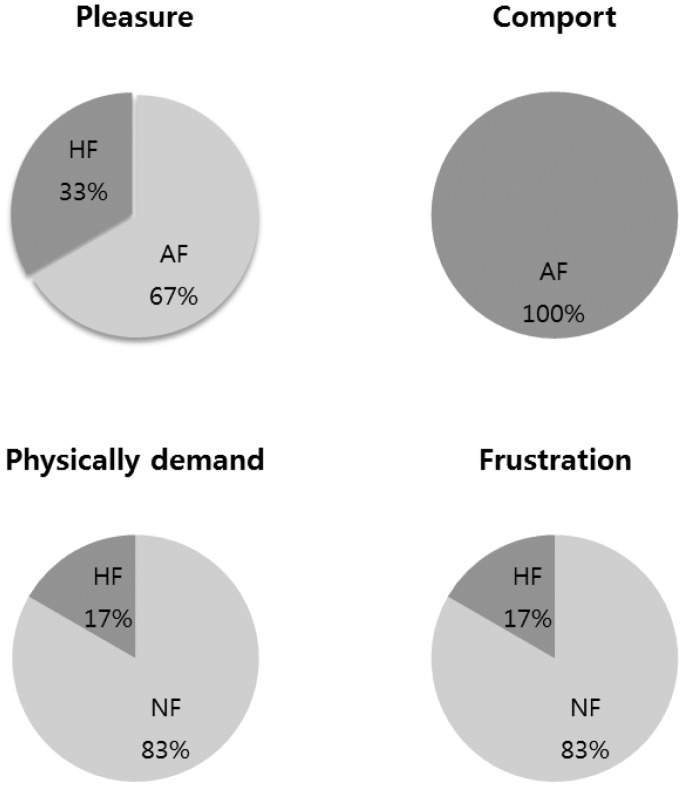
Participants' preferences about feedback conditions.

Figure 23 shows participants' preferences on feedback conditions. Overall, most of the participants said “a gesture interface without feedback is uncomfortable”. More than half selected audio feedback as the most pleasant feedback, while haptic feedback was chosen by about one third of the participants. All subjects said that audio feedback was most comfortable for the given gestures.

## 5. Conclusions

As introduced in [11], technologies of vision-based gesture interactions are being rapidly developed towards being more intuitive and natural, resembling human-to-human communications. This implies that better usability must be guaranteed when a new gesture control system is proposed and developed. In this aspect, we developed a new immersive hand gesture control system that employs both a novel hand tracking algorithm using a Kinect sensor and a high definition tactile feedback technology designed with a piezoelectric actuator for realistic mid-air gesture interactions. The developed 3D hand tracking algorithm is very accurate, robust against illumination changes and efficiently fast (maximum 12 ms) for being integrated with other independent modules, like gesture recognition and multimodal feedback. The evaluation results show that our vision-based tracking method outperforms other existing tracking methods. The average speed measured with the integrated system, including recognition and tactile feedback, was about 35 fps with an RGBD input image (320 × 240 pixels). Our developed system can also be used for many other applications, such as teleoperation, gesture-controlled immersive games, in-car driver interface, human machine interface, rehabilitation to monitor or train patients' motor skills, and so on.

Although there are many ways to provide tactile feedback to a user's hand, using a piezoelectric actuator is the most advanced technology, since it offers several benefits, such as high resolution temporal feedback, a fast response, light weight (thin) and strong vibration feedback. The prototype that we have developed with a thin (2 mm thick) piezoelectric actuator can be easily extended to other similar applications by minimizing the extra work in terms of software programming and modifying the haptic device. For instance, the prototype can be redesigned to drive multi-channel tactile feedback to the user's fingertips at the same time, which seems more realistic, as touching virtual objects displayed through a floating image display device. To the best of our knowledge, this is the first work that shows how to integrate tactile feedback into a vision-based hand gesture control system.

Our developed gesture control system efficiently works well in dynamic environments. To examine the performance of our system, a user study has been conducted with four basic gestures that can form any complex gestures. For the evaluation experiment, an existing gesture recognition algorithm, DTW-based gesture recognition [30], was implemented and combined with our gesture control system. The experimental results analyzed by a quantitative method, as seen in Figure 21, demonstrate that our gesture control system with tactile feedback is a promising technology for improving accuracy (higher recognition rates) and efficiency (shorter gesture trajectories) compared with the no feedback condition.

From a user experience perspective (usability), gesture speed and trajectory are considered major factors causing fatigue and discomfort, since it is clearly observed that: (1) from the quantitative experiment, tactile feedback or other feedback in gesture controls significantly affected the reduction of speed and trajectory compared to the no feedback condition; and (2) the analysis with the NASA TLX shows that all of the workloads were higher when users performed hand gestures with the no feedback condition. By closely investigating the results of the two experiments, it is found that speed and trajectory are closely related to the workload, because the data show that longer and the faster movements increase fatigue and discomfort. From this perspective, our study demonstrated that feedback can reduce fatigue and discomfort, which can eventually improve usability. One of the interesting findings is that haptic feedback can be a good solution to improve the mid-air gesture interactions in terms of performance and usability, although it is sometimes not the best.

One may argue why we focus on more haptic feedback than other feedback conditions that show similar or even better results. The answer would be that we focus on nonintrusive feedback that does not interfere with the purpose of the original content display. Additional visual and aural feedback may affect the original content display when operated by gesture controls. We therefore focus on analyzing the effect of tactile feedback while users control an external device with gestures. However, we were interested in comparing the system performance with all other feedback conditions to draw comprehensive and meaningful conclusions. For instance, Figure 23 suggests that aural feedback is the best option to improve comfort and pleasure. Our understanding for this result is that the prototype for the haptic feedback is not yet perfected to provide a comfortable interface as much as headphones do. This can be improved by redesigning the haptic device to be deformable for a better fit on the hand or by developing a bare hand touch feedback device, which is our ongoing research project.

We believe that the developed gesture interaction system must be a further step towards a natural user interface, though it has three limitations. One limitation is that our hand tracking algorithm works with two assumptions: no object exists within 10 cm of the user's hand and an acceptable moving distance (±15 cm) along the z-axis. In this study, the assumptions were made by testing with even more complex gestures. As the next step, we need to put more effort into developing an assumption-free algorithm. Another limitation is to use an elastic bandage to attach the tactile feedback device to the user's hand, which feels cumbersome to the user. This results from the flat surface design, which needs high pressure to have good contact with the user's palm surface. We can resolve this issue by redesigning the haptic device to have a deformable surface, so that users can change its shape for better attachment. We will work on this issue in the near future. The other limitation is the need to wear the device. Our haptic device is sufficiently light; however, wearing a device is still a burden for a natural user interface with bare hands. This problem can be solved by developing a non-wearable tactile feedback device, which is our ongoing research project. As the last future work, multimodal feedback effects on more complex gestures will be investigated by designing psychophysical experiments to understand the sensory dominance for mid-air gesture interactions.

## Figures and Tables

**Table 1.** Radius (r) of the MLBP used for the evaluation to measure the accuracy of hand detection at different distances.

**Distance (m)**	**1**	**1.5**	**2**	**2.5**	**3**	**3.5**	**4**	**4.5**	**5**	**5.5**	**6**	**6.5**	**7**
Radius (r)	125	85	65	55	45	40	35	30	25	25	25	20	20

**Table 2.** Radius values used for the hand tracking evaluation.

**Distance (m)**	**1**	**2**	**3**
Radius (r) of MLBP	40	30	20

**Table 3.** Averaged errors in the pixel and the standard deviations of our method in comparison with Natural Interaction Technology for End-user (NITE), 3D Hand Tracker with depth images and CamShift, at different distances (1 m, 2 m and 3 m).

**Distance (m)**	**1**	**2**	**3**
Proposed method	13.11 ± 2.37	8.48 ± 1.94	4.37 ± 1.20
NITE	16.59 ± 4.23	10.68 ± 2.64	5.21 ± 1.74
3D Hand Tracker	24.43 ± 9.56	20.26 ± 6.02	15.92 ± 4.27
CamShift	61.50 ± 20.37	45.55 ± 11.37	36.32 ± 8.93

**Table 4.** Averaged errors in the pixel of our proposed method in comparison with NITE, 3D Hand Tracker with depth images and CamShift, for different hand motions (circle, triangle, up to down and right to left).

	**Circle**	**Triangle**	**Up to Down**	**Right to Left**
Proposed method	7.93 ± 1.91	9.62 ± 2.43	5.73 ± 1.43	5.50 ± 1.52
NITE	9.60 ± 2.67	10.75 ± 3.24	7.35 ± 1.96	6.99 ± 1.93
3D Hand Tracker	19.52 ± 6.19	21.23 ± 9.27	17.87 ± 5.66	20.49 ± 6.98
CamShift	46.27 ± 10.15	52.90 ± 15.04	36.59 ± 9.98	36.16 ± 14.32
